# Supplementary material for: Alloying Iron into Palladium Nanoparticles for an Efficient Catalyst in Acetylene Dicarbonylation
Source: Nanomaterials (Basel). 2022 Oct 28;12(21):3803. doi: 10.3390/nano12213803 (PMC9654269; doi:10.3390/nano12213803)
Supplement: Supplementary file 1 [file nanomaterials-12-03803-s001.zip › nanomaterials-1928247-supplementary.pdf]

nanomaterials

Article

Alloying Iron into Palladium Nanoparticles for an Efficient Catalyst in Acetylene Dicarbonylation

Yuchen Zhang, Jianhui Zhang, Zongcheng Liu, Yiyi Wu, Yu Lv, Yadian Xie, Huanjiang Wang *

Key Laboratory of Low-Dimensional Materials and Big Data, School of Chemical Engineering, Guizhou Minzu University, Guiyang 550025, China
* Correspondence: whj2017@gzmu.edu.cn

Table S1. H_2-TPR results of reduced nano-Fe/Pd and Pd catalysts

catalyst	Amount of hydrogen consumed(μmol-H_2/g.)
nano-Fe/Pd	3721
Pd	5045
AgO	4315

Table S2. Catalyst ICP-OES elemental characterization

Sample	Pd wt.%	Fe wt.%
Fresh nano-Fe/Pd	69.8	3.8
First recycle nano-Fe/Pd	68.9	3.4
Fifth recycle nano-Fe/Pd	61.2	2.1
Fresh nano-Co/Pd	69.1	3.8
Fresh nano-Cu/Pd	70.7	4.2

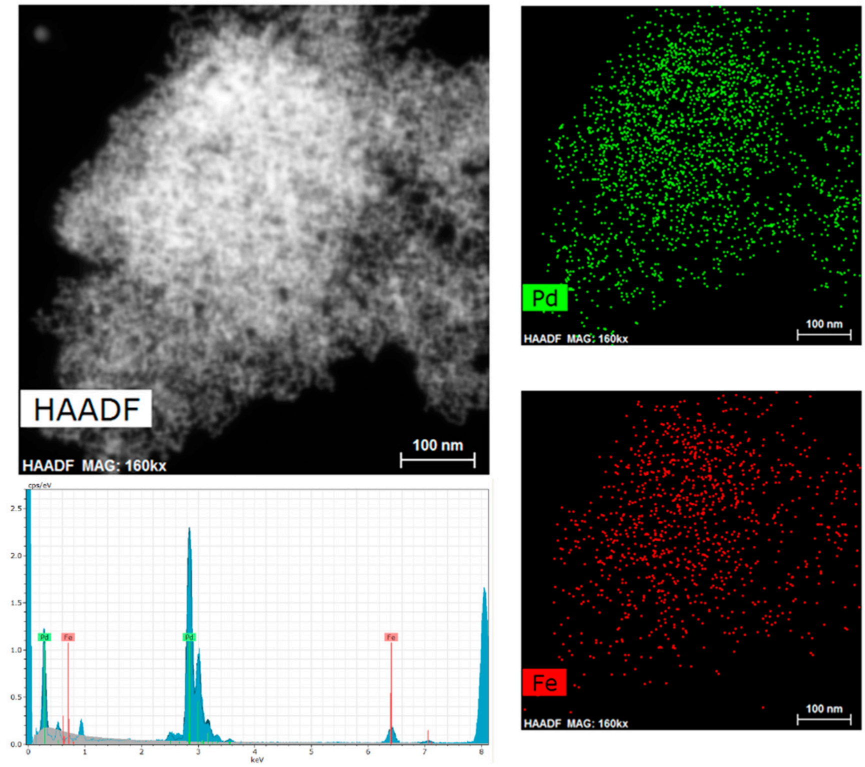

Figure S1. STEM-EDS elemental maps of nano-Fe/Pd

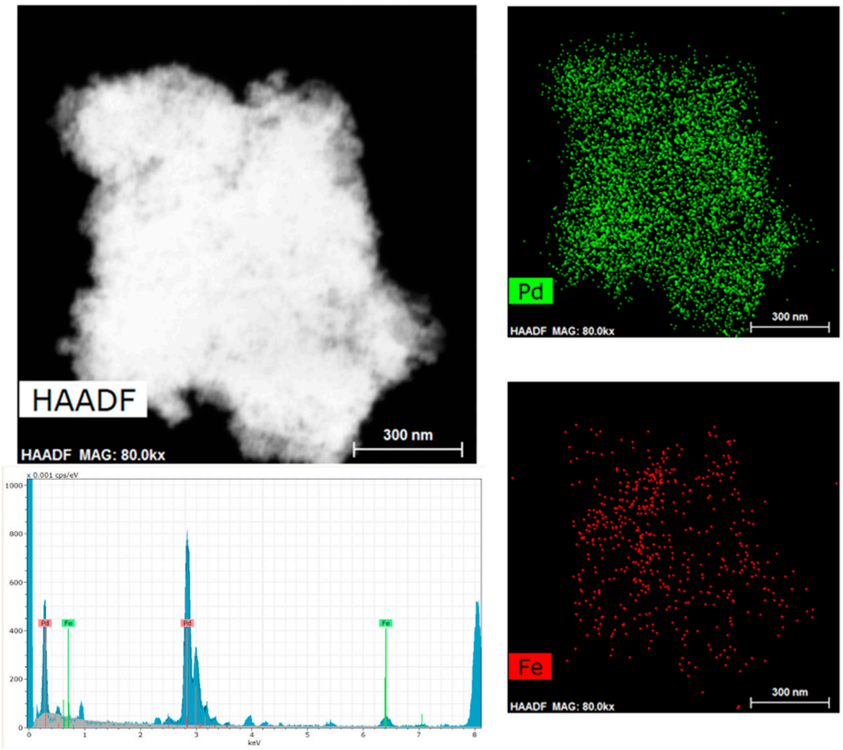

Figure S2. STEM-EDS elemental maps of fifth recycled nano-Fe/Pd

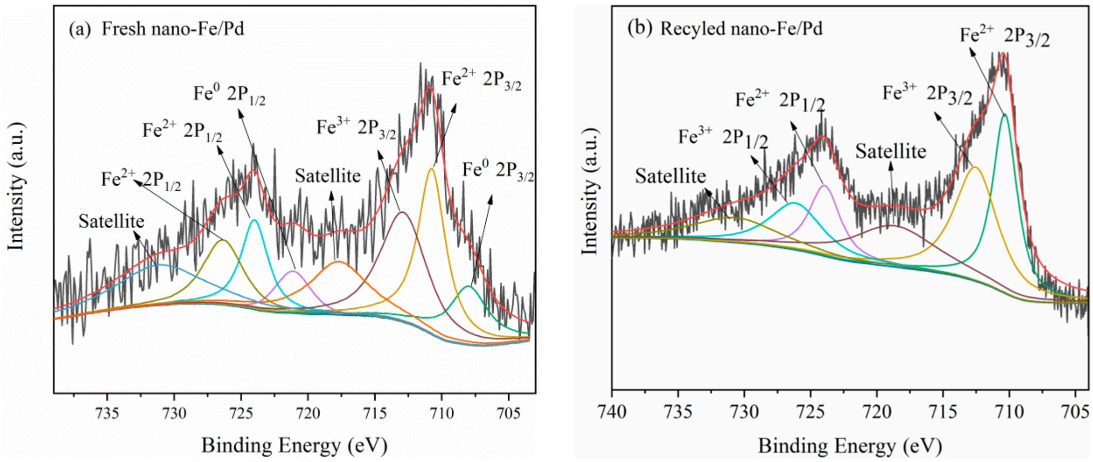

Figure S3. High-resolution Fe 2p XPS spectra of fresh nano-Fe/Pd catalyst (a) and fifth recycled nano-Fe/Pd catalyst (b)

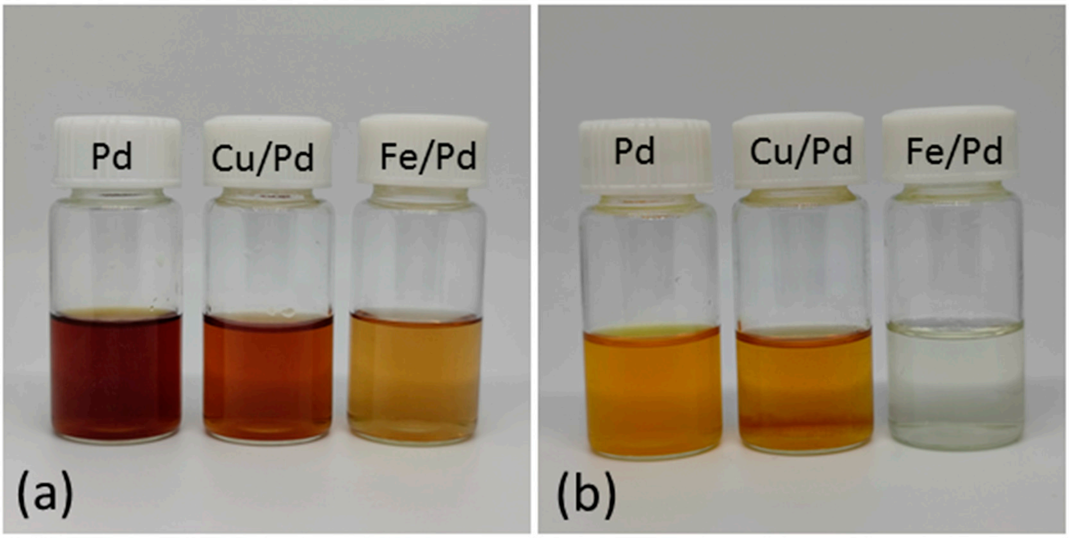

Figure S4. Supernatant color of the reaction system before (a) and after (b) treating with $K_2S_2O_3$

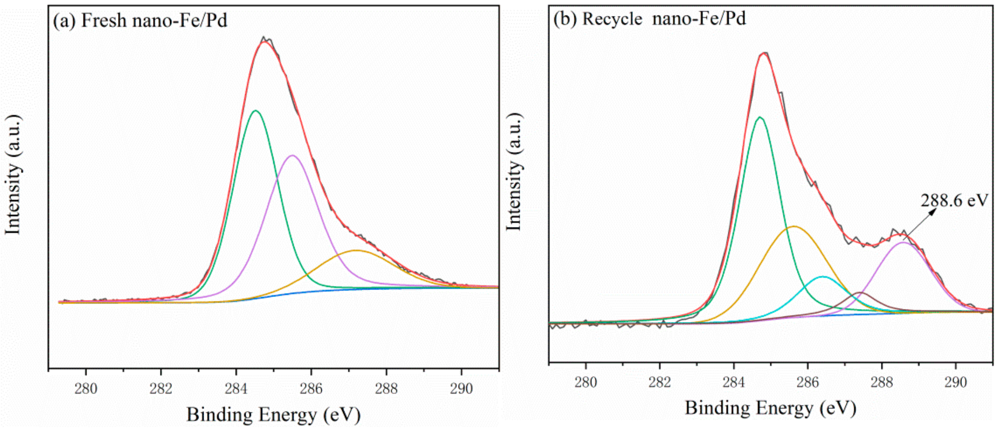

Figure S5. High resolution C 1S XPS spectra of fresh nano-Pd (a) and five recycled nano-Fe/Pd (b)

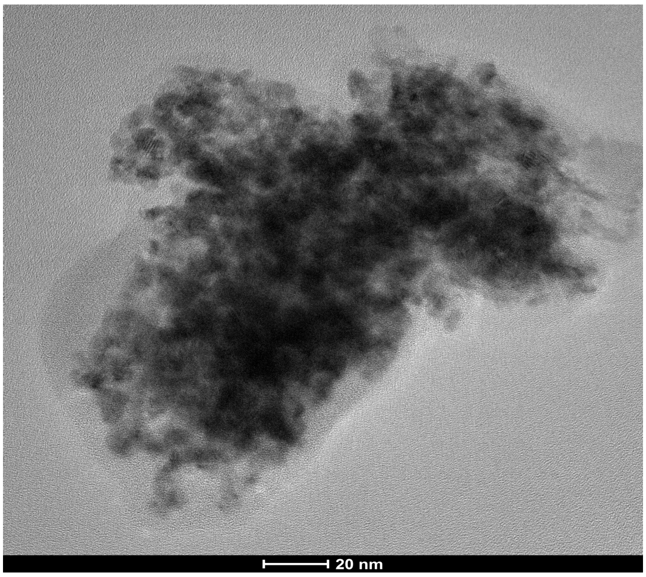

Figure S6. TEM images of fifth recycled nano-Fe/Pd

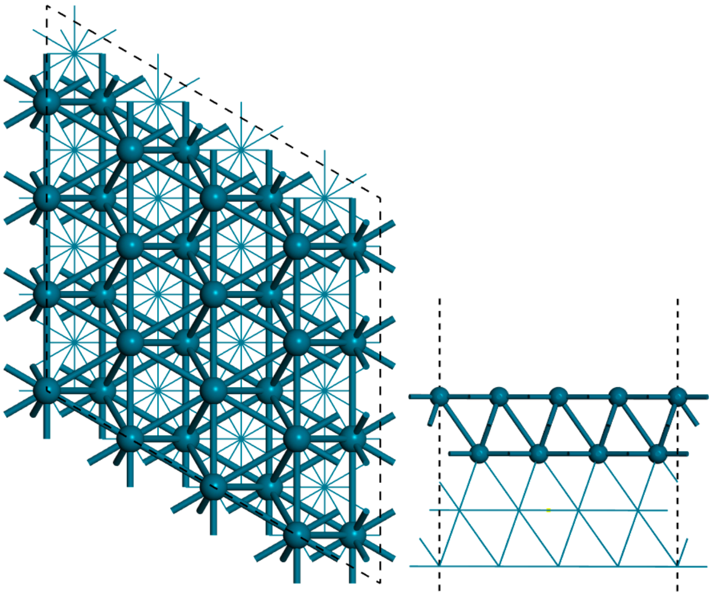

(a) Pd(111)

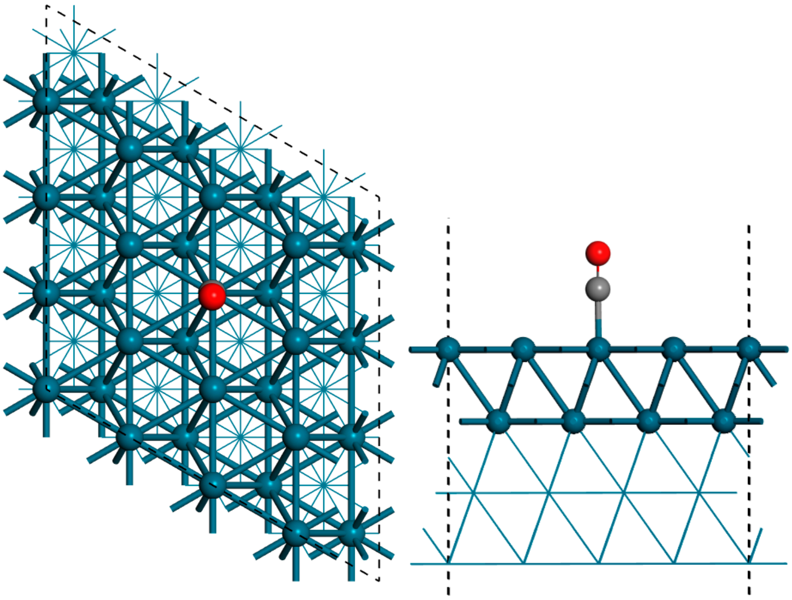

(b) Pd(111)-CO-top

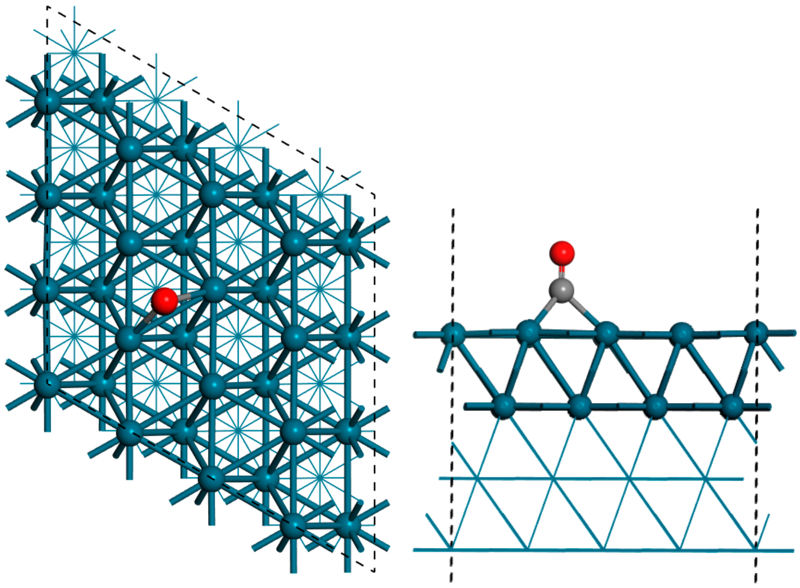

(c) Pd(111)-CO-bridge

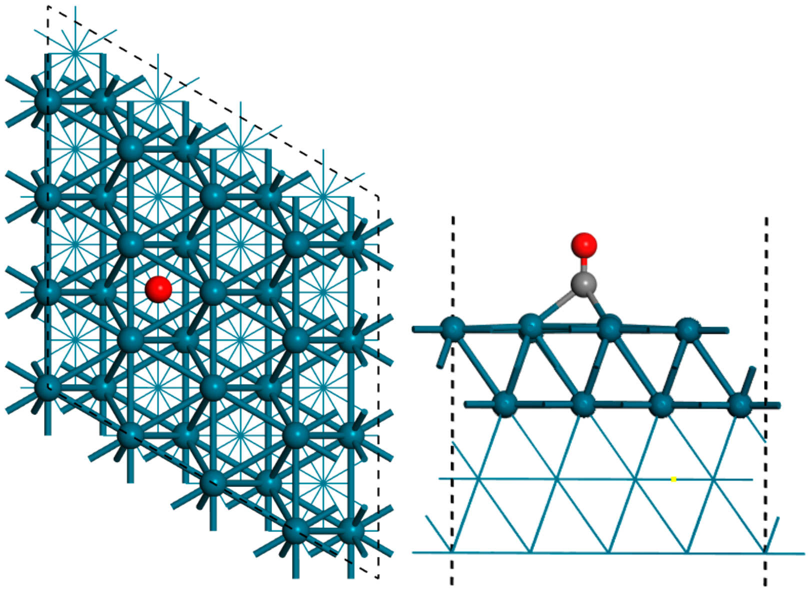

(d) Pd(111)-CO-hcp

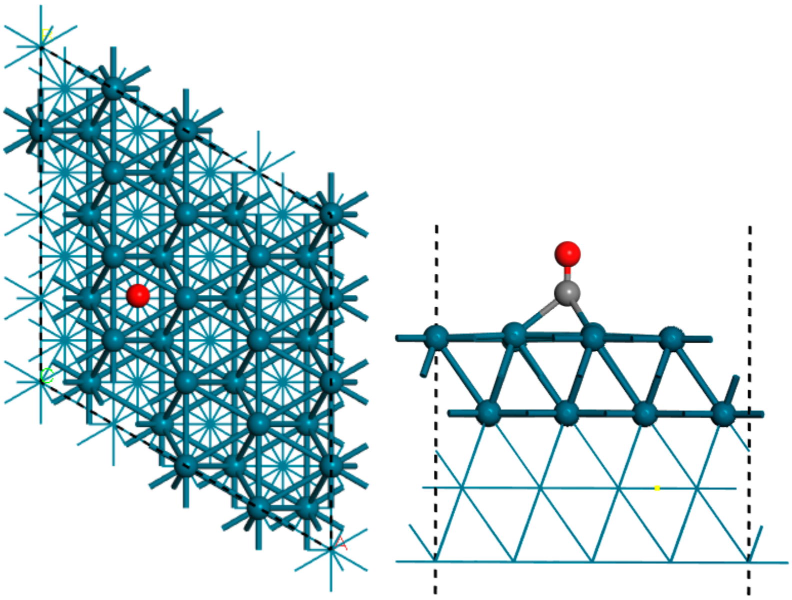

(e) Pd(111)-CO-fcc

Figure S7. DFT optimized structures of Pd(111) and different CO adsorption configurations on Pd(111) and the detialed explanations of each configrations given by subtitle letters (**a-e**)

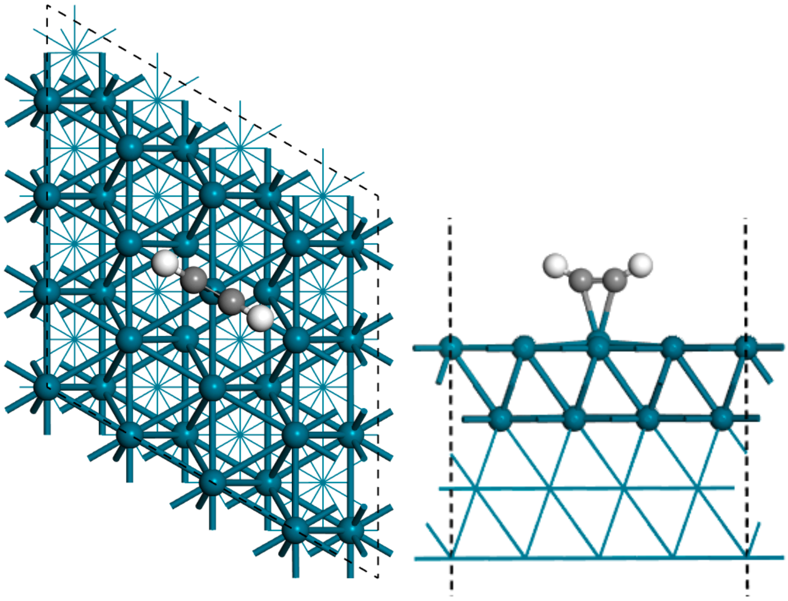

(a) Pd(111)-C_2H_2-Top

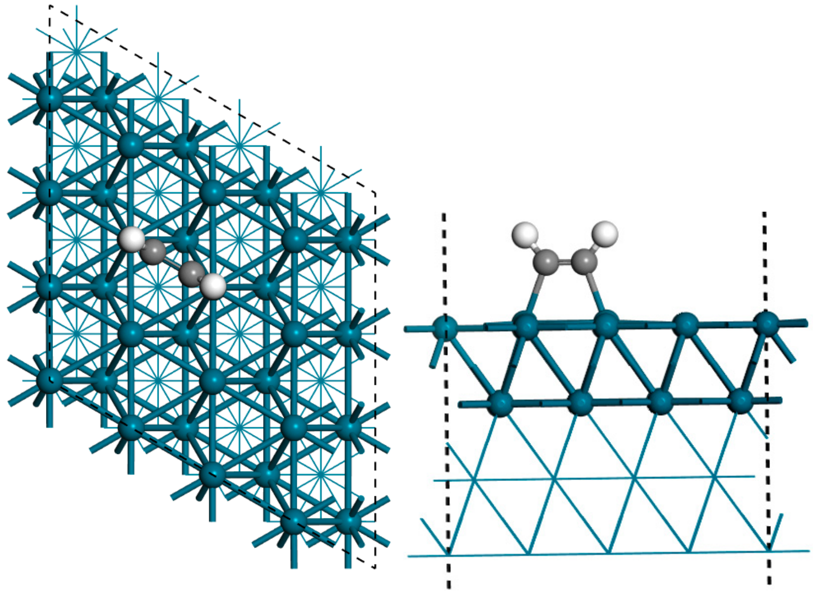

(b) Pd(111)-C_2H_2-Bridge

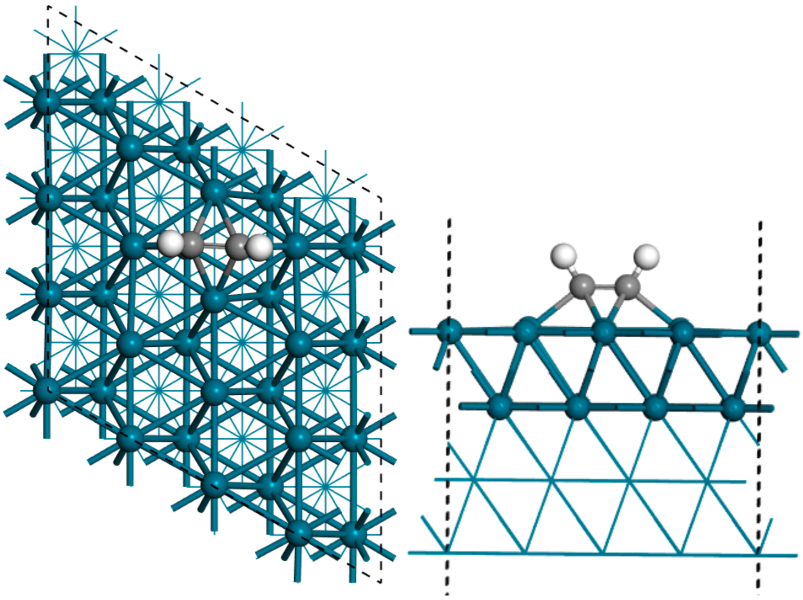

(c) Pd(111)-C_2H_2-Bridge-2

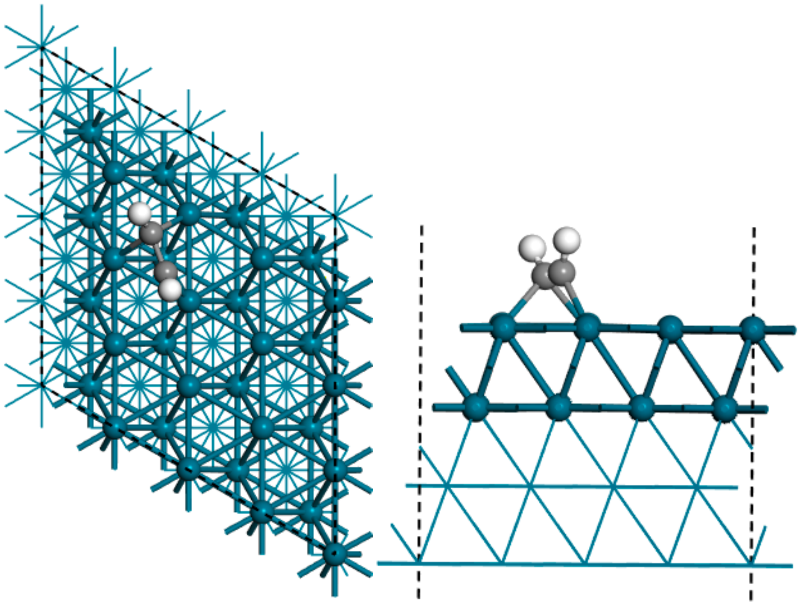

(d) Pd(111)-C_2H_2-hcp

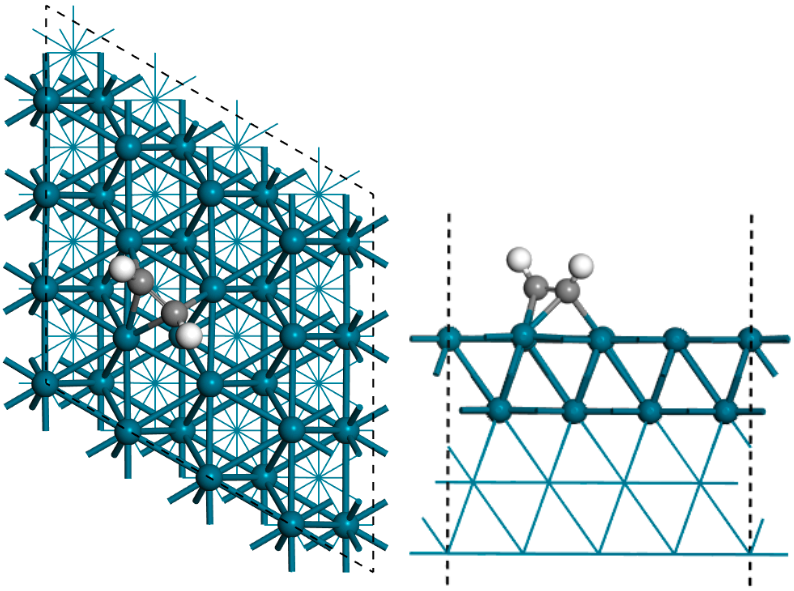

(e) Pd(111)-C_2H_2-fcc

Figure S8. DFT different optimized structures of C_2H_2 adsorption configurations on Pd(111) and the detialed explanations of each configrations given by subtitle letters (**a-e**)

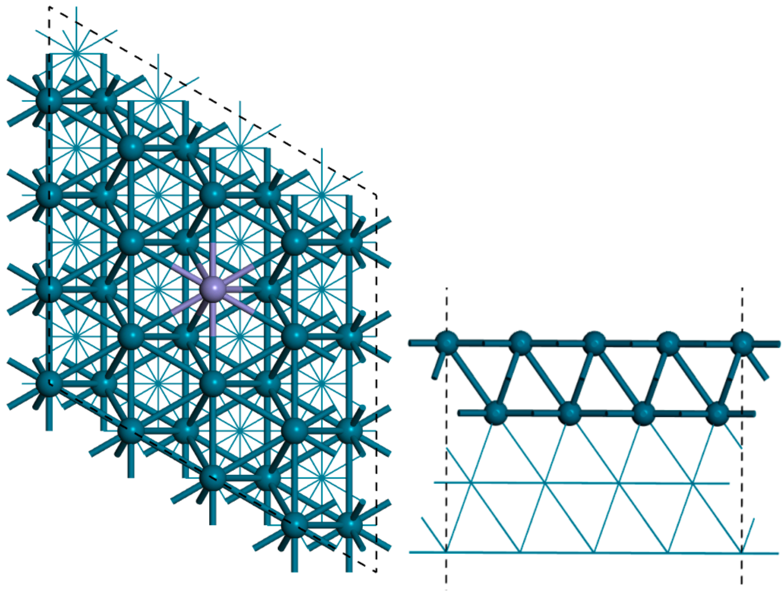

(a) Fe/Pd(111)

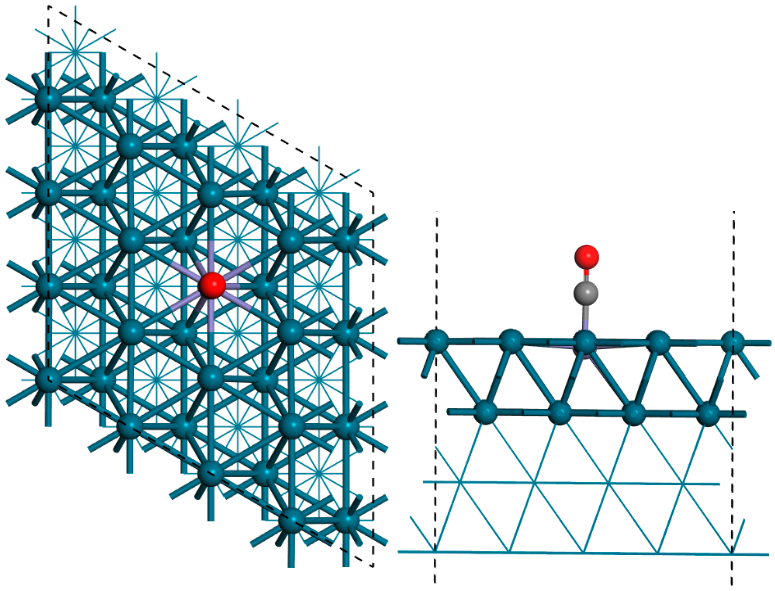

(b) Fe/Pd(111) -CO-top

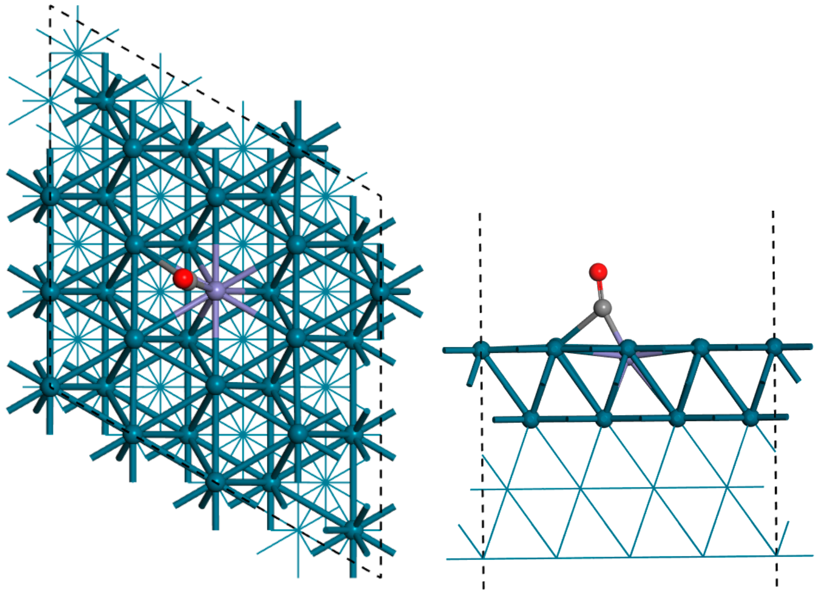

(c) Fe/Pd(111) -CO-Bridge

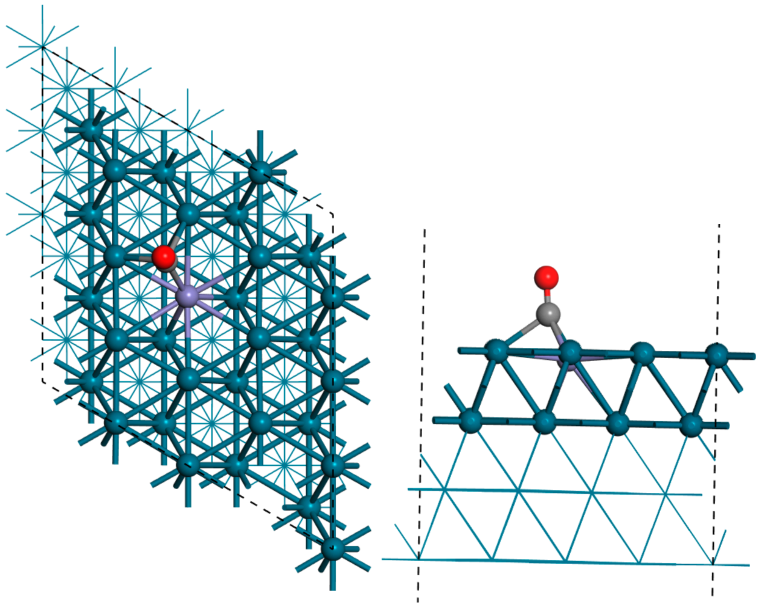

(d) Fe/Pd(111) -CO-hcp

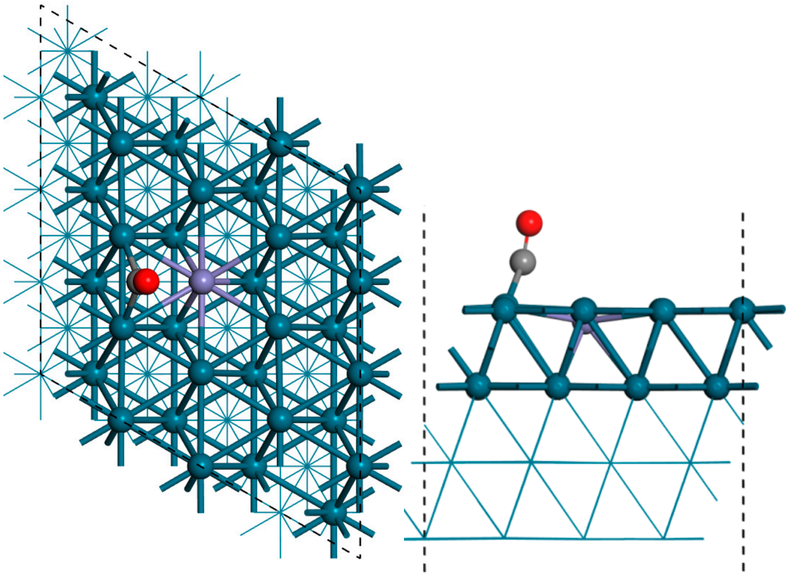

(e) Fe/Pd(111) -CO-fcc

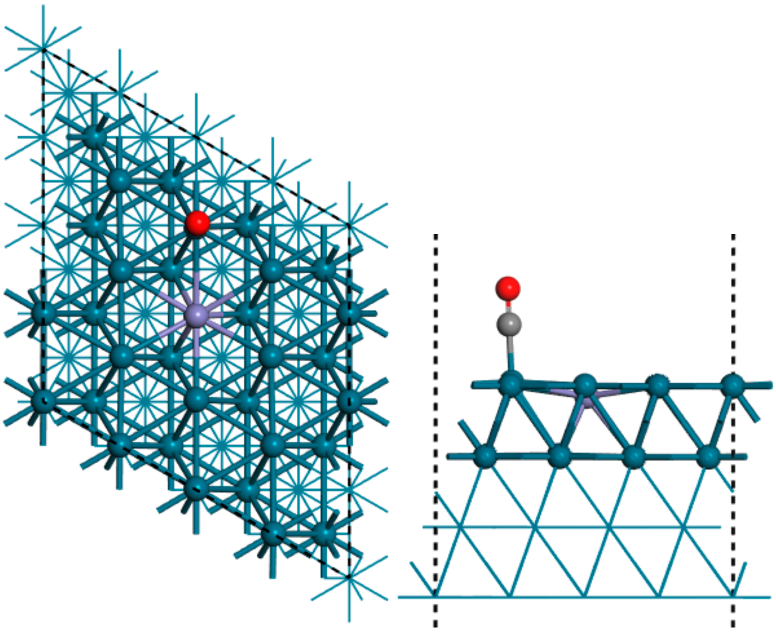

(f) Fe/Pd(111) -CO-top-2

Figure S9. DFT optimized structures of Fe/Pd(111) and CO adsorption configurations on Fe/Pd(111) and the detialed explanations of each configrations given by subtitle letters (**a-f**)

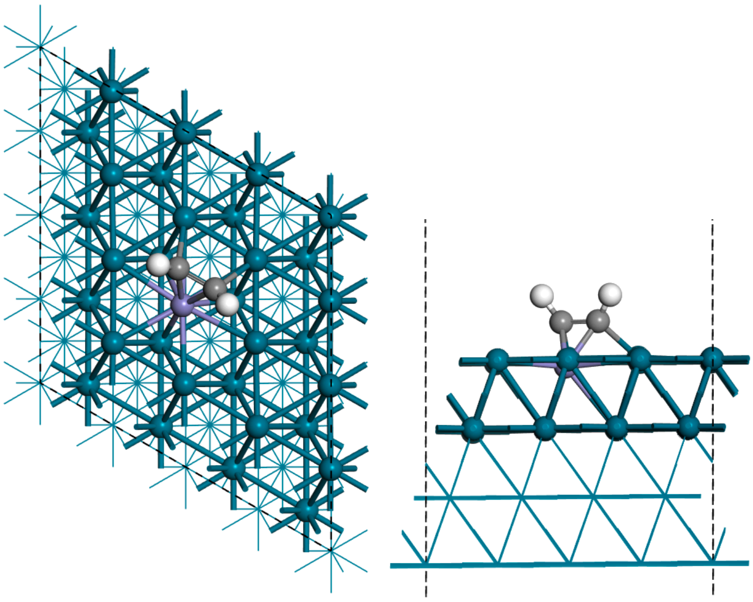

(a) Fe/Pd(111)-C_2H_2-Top

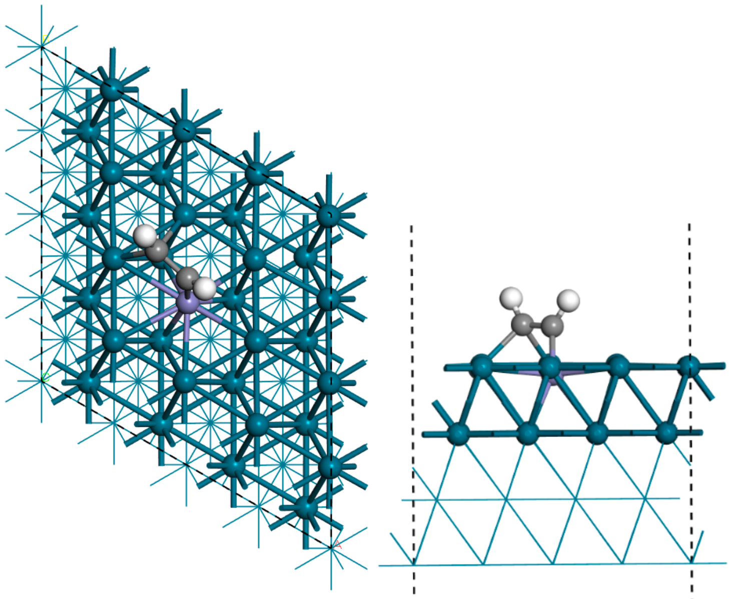

(b) Fe/Pd(111) -C_2H_2-bridge

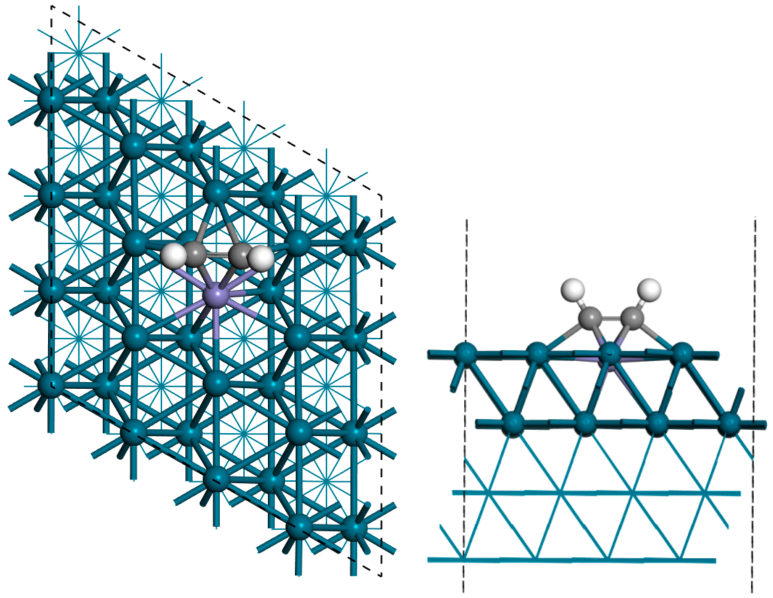

(c) Fe/Pd(111) -C_2H_2-bridge-2

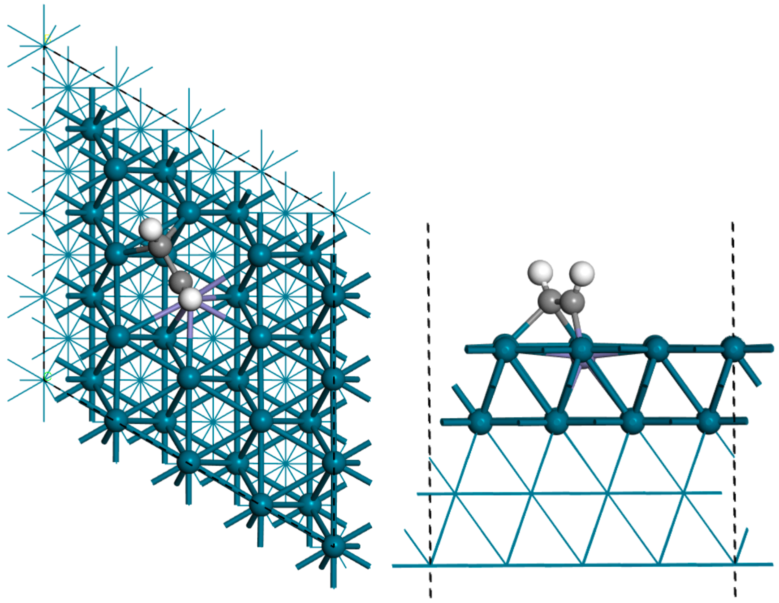

(d) Fe/Pd(111) -C_2H_2-hcp

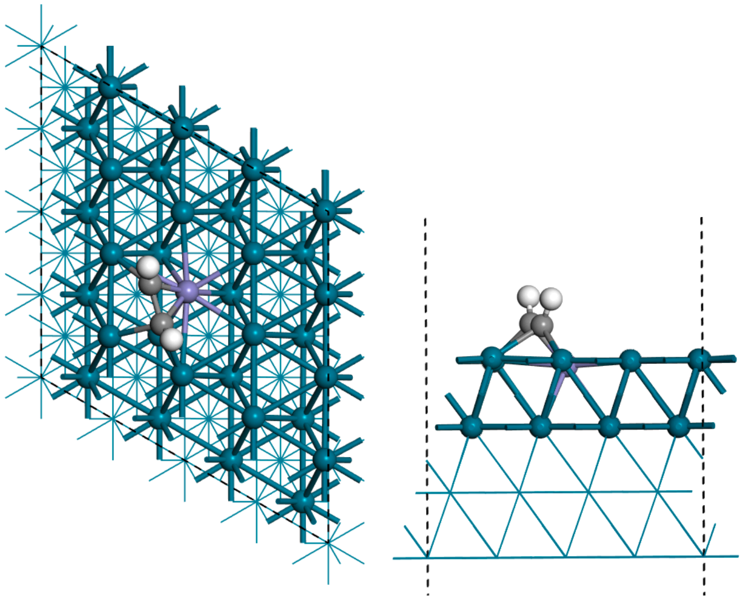

(e) Fe/Pd(111) -C_2H_2-fcc

Figure S10. DFT different optimized structures of C_2H2 adsorption configurations on Fe/Pd(111) and the detialed explanations of each configrations given by subtitle letters (**a-e**)

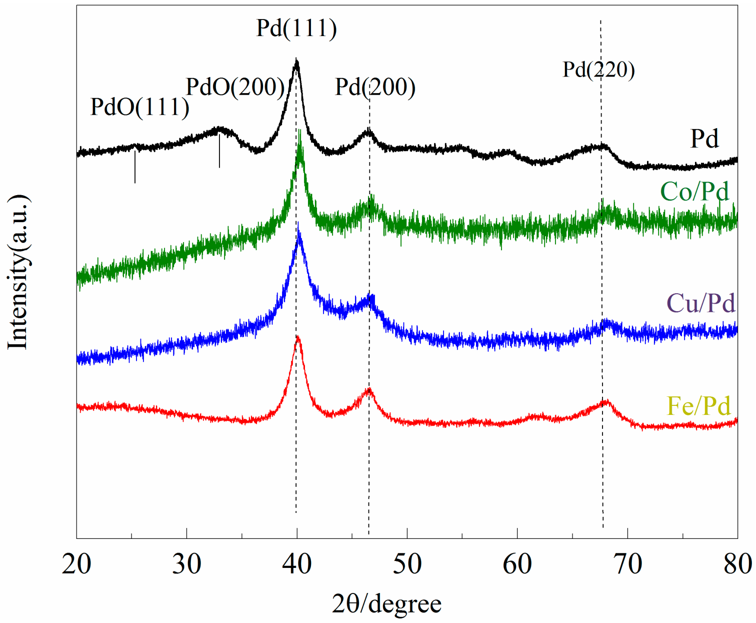

Figure S11. XRD patterns of nano-Fe/Pd, nano-Cu/Pd, nano-Co/Pd and nano-Pd samples

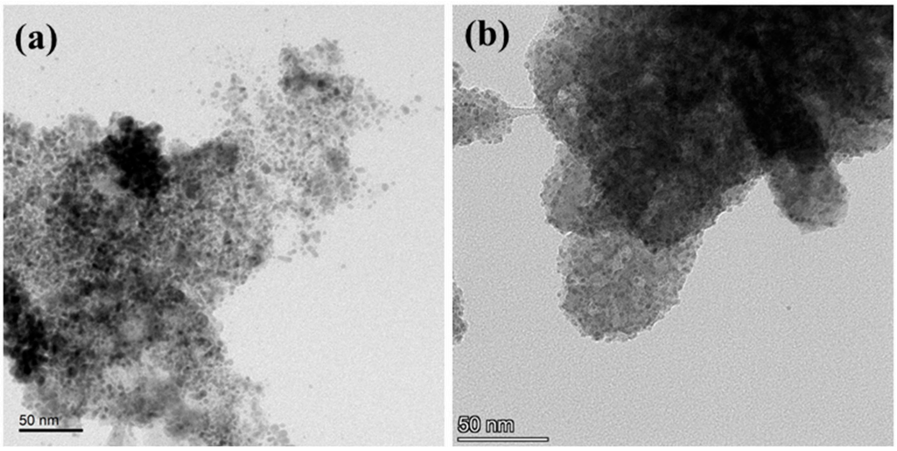

Figure S12. TEM images of nano-Co/Pd (**a**) and nano-Cu/Pd (**b**) nanoparticles
